# Exploring Self-Care, Anxiety, Depression, and the Gender Gap in the Software Engineering Pipeline

**DOI:** 10.3390/ijerph21111468

**Published:** 2024-11-04

**Authors:** Alicia Julia Wilson Takaoka, Letizia Jaccheri, Kshitij Sharma

**Affiliations:** 1 Department of Computer Science, Norwegian University of Science and Technology (NTNU), 7034 Trondheim, Norway; letizia.jaccheri@ntnu.no (L.J.); kshitij.sharma@ntnu.no (K.S.); 2 Rotterdam School of Management, Erasmus University Rotterdam, 3062 Rotterdam, The Netherlands

**Keywords:** burnout, mental health, software engineering pipeline, social justice, anxiety, depression, gender gap, age

## Abstract

Software engineers may experience burnout, which is often caused by the anxieties and stresses of the workplace. Understanding the well-being and resilience practices of software engineers and evaluating their knowledge of mental health is one factor to understand our current, diverse, multi-generational workplaces. Here, we present preliminary results of a study examining the self-care practices of software engineers, a general overview of the state of mental health of software engineers, and correlations between expressions of mental health and demographic factors. Among 224 respondents, positive correlations between imposter syndrome and happiness, anxiety, and depression were identified. We also identified negative correlations between mental health literacy and imposter syndrome, happiness, anxiety, and depression. Well-being had a positive correlation with self-efficacy, as well as with happiness. We also present the Gender Gap in mental health and our findings in relation to that construct. Our findings suggest increasing mental health support services.

## 1. Introduction

The built environment is designed by software engineers, so software engineers wield the power to affect change for users, customers, or those of varying ages and backgrounds that use technology. This is because users participate in built environments, use web- or mobile-based tools and applications, and incorporate software in their daily lives, whether willingly or unwillingly. The built environment is a collection of industries that are extremely competitive spaces because of the economic impact of software, and particularly AI, both of which are billion-dollar industries (https://www.bloomberg.com/company/press/generative-ai-to-become-a-1-3-trillion-market-by-2032-research-finds/ accessed on 6 June 2024). The pressure to succeed as a programmer begins early through recruitment and during the application process to get into competitive universities [1] and persists throughout one’s career [2] as time pressure [3] and pair-pressure [4] are learned behaviors that are taught in college classes. These persist and are compounded with budget and schedule pressures in the workplace [5]. As a result, examining the well-being of software engineers, both as students and in the workplace, is becoming imperative.

The path to becoming a software engineer begins well before college, with research showing activities in elementary school can foster computational thinking as well as hinder or facilitate one’s choice to study computer science (e.g., [6,7]). In addition, mental health issues, particularly persistent problems, are not alleviated by changing roles from student to employee. This is partially because the transitions that occur between the ages of 18 to 29 are considered emerging adulthood [8], a new life stage that has notable implications for career and mental health because of the transitional and volatile nature of personal development [9]. In addition, sense of belonging in the field of software engineering, both as a student [10] and in the workplace [11], is in-part derived from participating in causes that promote positive change or social good, particularly for women [12].

Social justice issues, like the transition to climate neutrality, gender balance, receiving a quality education, or other issues related to the UN Sustainable Development Goals (https://sdgs.un.org/goals accessed on 6 June 2024) are areas that are significantly impacted by software development. Thus, the well-being of software developers throughout the software engineering pipeline, particularly in college and in the workforce, should be examined to understand the impacts a career in software engineering, and particularly social justice software development, has on mental health. In this paper, we explore the concepts of well-being, resilience, self-care, burnout, and mental health in relation to people in different phases of their software development career.

A clarification of the terms we use is necessary for understanding the contributions of software engineers to the built environment. Well-being and resilience are often seen as conflated characteristics. Well-being is considered a composite construct comprised of six characteristics that impact how a person perceives and interacts with the world around them. The characteristics that make up well-being are environmental mastery, personal growth, purpose in life, autonomy, self-acceptance, and positive relations with others [13]. Resilience is often seen as “the capacity to maintain or recover high well-being in the face of life adversity” [13]. Resilience is a necessary component for software developers in the built environment who deal with high-stress social justice issues like the transition to climate neutrality. Self-care is “the care of oneself without medical, professional, or other assistance or oversight” (Albert, 2008, in [14]), and self-care practices are the activities a person performs to promote that type of independent care, while self-efficacy is related to self-directness or the ability to depend on oneself to be responsible, purposeful, and resourceful [13]. In this short paper, we review the connections between self-care, mental health literacy, and the Gender Gap to better understand the resilience and needs of software developers in the built environment.

## 2. Background

Quantifying and monetizing emotions has been effective in showing the value of the mental heath, well-being, and resilience of employees in the workplace. This information can be used to influence workplace policies and change toxic environments [15]. A generally agreed upon definition for happiness in software developer research is experiencing more positive affective emotions than negative affective emotions [16]. While there is some debate about whether people choose to be happy or not, the debate about happiness in the workplace shows that happiness is a determining factor in productivity. In software development, productivity is shown in the quantitative values for pushes, pulls, and commits [17], and when a software developer’s self-reported perception of happiness is high, they tend to be more productive in the quantitative terms. These are indirect ways to evaluate the mental health of software developers.

Mental health awareness and the adoption of mental health literacy has increased since the COVID-19 pandemic. Jorm defined mental health literacy as the “knowledge and beliefs about mental disorders which aid their recognition, management or prevention” [18]. Jorm also proposed that mental health literacy involves knowing how to prevent mental disorders, being able to recognize when a mental disorder is developing, knowing about help-seeking options and treatments available, knowing about self-help strategies, and mental health first-aid skills to support others affected by mental health problems [18]. To assess mental health literacy, we adopted the Australian Mental Health Literacy Survey by Bakker et al. [19]. Mental health literacy is only one aspect of the mental health factors impacting software engineers.

### 2.1. Mental Health Factors in Software Engineering Research

Quantifying and monetizing emotions has been employed to show the value of the mental health, well-being, and resilience of employees in the workplace. This information can be used to influence workplace policies and change toxic environments [15]. An often used definition for happiness in software engineering (SE) research is experiencing more positive affective emotions than negative affective emotions [16]. While there is some debate about whether people choose to be happy or not, the debate about happiness in the SE workplace shows that happiness is a determining factor in productivity. In SE, Smite et al. [17] and Graziotin et al. [16,20,21] show productivity as the quantitative values for pushes, pulls, and commits [17], and when a software engineer’s self-reported perception of happiness is high, they tend to be more productive in the quantitative terms.

**Figure 1:**
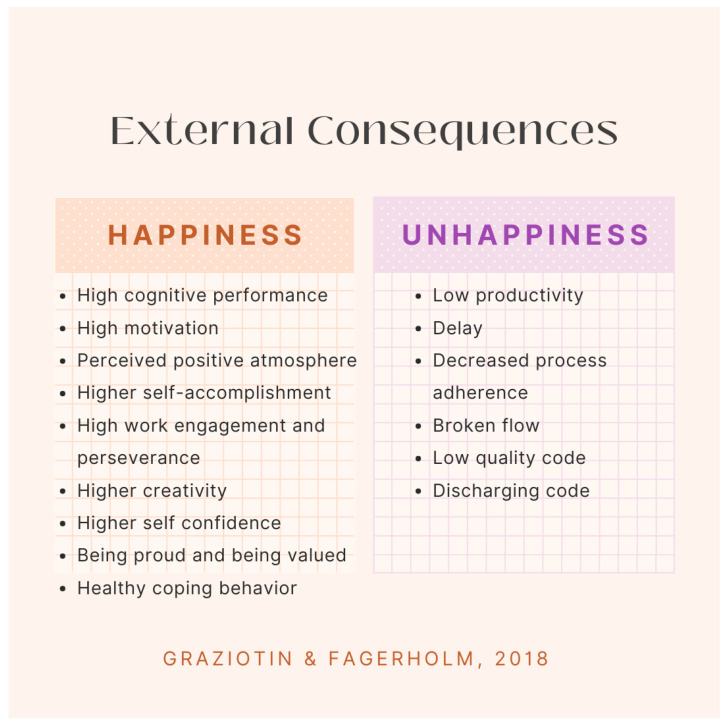
According to the research of Graziotin and Fagerholm [16], a list of external consequences of happiness in the software engineering workplace is identified.

The effects of happiness and unhappiness in SE are now starting to be evaluated. Graziotin and Fagerholm [21] led a series of studies that evaluate the emotional states of happiness and unhappiness and their impacts on software development. In a survey of 664 respondents, researchers identified several internal consequences, or those that affect the developer, that are associated with happiness and unhappiness in the workplace. They are in Figure 1.

**Figure 2:**
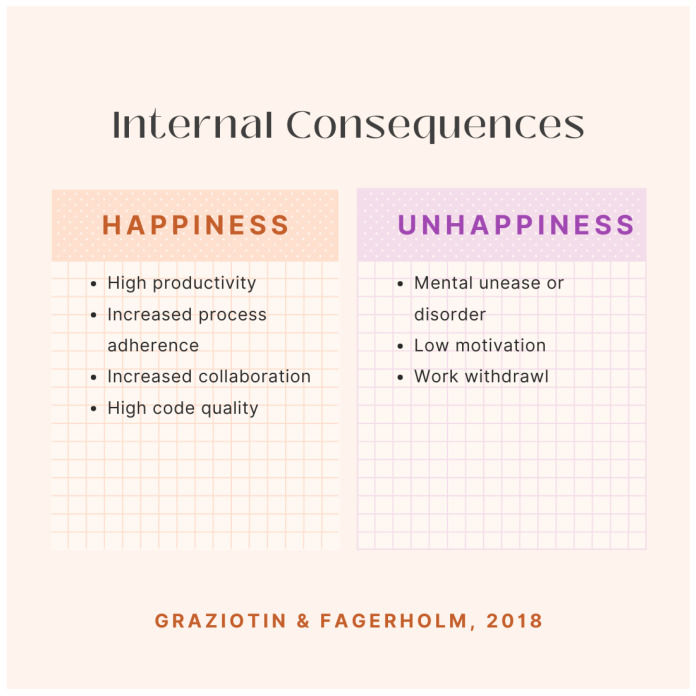
According to the research of Graziotin and Fagerholm [16], a list of internal consequences of happiness in the software engineering workplace is identified.

As shown, there are more positive consequences found from happiness than there are negative consequences found because of unhappiness in the workplace. This is also true for internal consequences, as seen in Figure 2.

In addition, studies on software quality have indicated that there is a positive relationship between happiness and other aspects of performance. Graziotin et al. state many studies have shown links between affect, emotions, politeness, and the quality of software designed. The authors also point to studies that show reductions in burnout and anxiety when perceived happiness is high [16]. To assess these mental health qualities, we used the Beck Anxiety Inventory (BAI), Beck Depression Inventory (BDI), Warwick–Edinburgh Well-Being Scale, the Coping Self-Efficacy Scale, the Australian Mental Health Literacy Survey by Bakker et al. [19], the Clance Imposter Phenomenon Scale, and the Oxford Happiness Index. It can be speculated that burnout in tech fields can be higher when a person experiences more negative consequences.

### 2.2. Burnout

Burnout in tech is high [17]. Because burnout is related to anxiety, and unhappiness is related to anxiety, examining connections in mental health symptoms, awareness, and practices may reveal information about the current state of the mental health of software engineers and their needs. These connections may lead to a better understanding of the well-being of software engineers in digital justice, or technology to better society in some way, and may experience compounding factors of tech burnout and social justice burnout.

Social justice burnout is a type of burnout from being in and engaging in situations that are physically, mentally, and emotionally demanding. Social justice burnout is different from other types of burnout because it includes the added burdens of engaging with triggers, engaging with past trauma, and working directly or tangentially with marginalized individuals or groups who have been wronged in a serious way and are seeking reparations or healing [22]. Being constantly confronted with societal issues in the pursuit of justice or change may cause one to leave activism or abandon their idealism [22]. The burden caused by this social justice burnout and the associated digital justice work, which we define as tech that directly impacts the lives of users for social betterment and IDEA (Inclusion, Diversity, Equity, and Accessibility), is omitted from research about software developers in the built environment.

### 2.3. Self-Care in Software Engineering

The impacts of working from home on happiness and productivity in software engineering have been evaluated as well. Smite et al. [17] examined the pull, push, commit, and code reviews both pre-pandemic and during the pandemic on GitHub and found a slight increase during the pandemic year 2020. In addition, the researchers found that most workers also reported increased job satisfaction when working from home as opposed to going into the office. Still, the change in locale does not mitigate inter-generational interactions and needs in the workplace.

This is the most diverse workforce in history, with five generations dealing with nuances and changing norms, and software engineering is no different. The growing awareness of mental health differences and needs in the workplace has sparked investigation into who is experiencing challenges, what those challenges are, and possible mitigation or intervention factors. Growing areas of interest about software developers and mental health are in anxiety [21], depression [23], and the imposter phenomenon, also called imposter syndrome [24].

Research about self-care in software engineering is still emerging. While several articles examine software and applications developed to facilitate or prioritize self-care [25,26], at the time of writing, only one article found examined affective emotions in software engineers [27]. Penzenstadler explores how mindfulness, yoga, and breath work practices may impact software engineers and, by proxy, their work. Using creativity theory, a card deck, toolkit, and training framework for both classroom and workplace were developed. These interventions may help reduce anxiety and depression in software engineers. Collectively, the research about self-care and burnout has sparked research in the area of the Gender Gap in mental health. The Gender Gap in depression states that women report higher rates of depression than men.

### 2.4. Gender Gap in Mental Health

Research on the Gender Gap in depression has been ongoing throughout the 2000s. McDonough and Strohschein [28] found that more women report experiencing depression than men and that this increases with age. Corroborating these findings, Acciai and Hardy [29] sought to identify causes for why this Gender Gap, particularly in reporting depression, exists. They found that sample composition, differences in reporting, and strategies in reacting to adversaries may contribute to the Gender Gap. In order to examine whether this Gender Gap in depression was changing over time, Platt et al. [30] conducted a meta-regression analysis on a sample of studies from the United States from 1980 to 2019. They found that there was no change in the Gender Gap for depression in adults, indicating that factors such as education and socio-economic status do not impact depression. Bracke, Delaruelle, Dereuddre, and Van de Velde [31] found that in 29 European countries, the Gender Gap exists but also increases with age. Finally, Mirowsky and Schieman [32] found the Gender Gap to also exist in anxiety and anger reporting and that the Gender Gap for anxiety also increases with age.

In all of the above studies, the Gender Gap in anxiety and depression increases in old age and is higher for women than men. While no correlation between these sets of findings and resilience or self-care exists, research on mental health factors, burnout, self-care, and the Gender Gap in depression has led to the development of the following research questions:

- **RQ1:** What are the common themes that emerge for software engineers about mental health?
- **RQ2:** To what extent are the states of mental health in alignment between students and practitioners in software engineering?
- **RQ3:** What is the state of the Gender Gap in anxiety and depression in software engineers today?

We use these research questions to guide our evaluation of the mental well-being of software engineers at various stages and occupying different roles in the software engineering pipeline to better understand their impact on designing the built environment.

## 3. Methodology

This is a correlational study, which means the data are unmanipulated for analysis. Based on the results of a systematic literature review in which tools and assessments for evaluating the status of mental health among computer science students were identified, we constructed a survey to investigate any possible connections between the self-reporting of mental health, self-care, and career or field of study. The survey was classified as exempt from the ethics review board based on the anonymity of respondents at the point of data collection.

### 3.1. Instrument Design

The survey consisted of demographic information, open-ended questions relating to self-care, and several instruments to evaluate the state of mental health. The survey included demographic questions about age, education, political affiliation, gender, a multi-select option for role (Advocate, Analyst, Consultant, Designer, Developer, Doula, Educator, Engineer, Manager, Programmer, Researcher, Student, Tester, UX, User, and Other), open-ended questions about professional field (like informatics, data science, and fintech), and where participants live and work. Political alignment and other characteristics were collected because these values shape responsible tech design, development, use, and ethics.

The questions relating to self-care sought to gather perceptions about the importance of self-care in general, as well as how it applies to the self. We also sought to capture thoughts and attitudes about the relationship between work and its impact on self-care. These questions were followed by a series of instruments, all used with licensing permissions for research and academic use. The instruments used were the Warwick–Edinburgh Well-Being Scale, The Coping Self-Efficacy Scale, the Mental Health Literacy Survey, the Clance Imposter Phenomenon Scale, and the Oxford Happiness Index.

### 3.2. Population

Graziotin defines a software developer as “a person concerned with any aspect of the software construction process for any purpose such as work, hobby, or passion” ([16], p. 34). We adopt that definition and extend it to include students for the purpose of this study. Participants were selected based on their involvement in the software engineering pipeline at any stage as an entry point. We are interested in investigating the pipeline as a whole since many studies only examine either students, practitioners, or users. This follows the approach seen in Gralha et al. [33], Kohl et al. [34], and Motogna et al. [35] in which populations are enmeshed in order to perform a more thorough analysis across age and experience.

Two distinct groups, university students in computing programs and practicing software engineers, were targeted. We solicited responses from Mechanical Turk, institutional listservs in Norway and the United States, and Slack channels dedicated to practitioners in AI and tech development. Students and practitioners are targeted in this study because many students work and participate in software engineering processes and environments throughout the course of their studies. This can be through internships, consulting projects, contributing to open-source communities like GitHub and Wikipedia, and even direct full- or part-time employment with companies. In addition, emerging adulthood spans from, late teens to late 20s, and during this time, many adults face difficulties in transition, whether from living with parents to university, university to work, or other personal moments of transition. Our estimated population is approximately 47,300 practitioners in the software engineering pipeline.

### 3.3. Data Collection

The survey was disseminated to two Slack communities of tech professionals who work in SE. It was also disseminated to university listservs for students in computer science to complete and share. We also opened submissions on Mechanical Turk to gather the perspectives of people who use computers for work. The survey was open and collecting responses from 14 September 2023 to 14 October 2023.

We received a total of 284 responses. Of those, 57 were omitted due to duplicate answers generated by an AI response. These responses did not accurately address the questions asked. We used the questions related to current field of employment and providing a definition of self-care as a barometer for survey response accuracy.

### 3.4. Data Analysis

The demographic information was evaluated using descriptive statistics. Instruments were analyzed using the methods outlined in the licensing agreements for calculating scores and subscores. In addition, alluvial plots, mediation regression, and Pearson’s correlation were employed to evaluate the connections between self-reported mental health status and composite scores of each instrument representing well-being and resilience. For comparing mental health literacy and an interest in working with digital justice, an unpaired *t*-test was conducted.

For comparing anxiety and depression across gender and age groups, we used one-way ANOVA with Welch correction for non-equal variances in different groups. We also used interaction effect analysis to understand the interplay of age and gender and its relationship with anxiety and depression. For checking the dependence between age and gender, we used the chi-square test and analyzed the residuals.

## 4. Results

**Figure 3:**
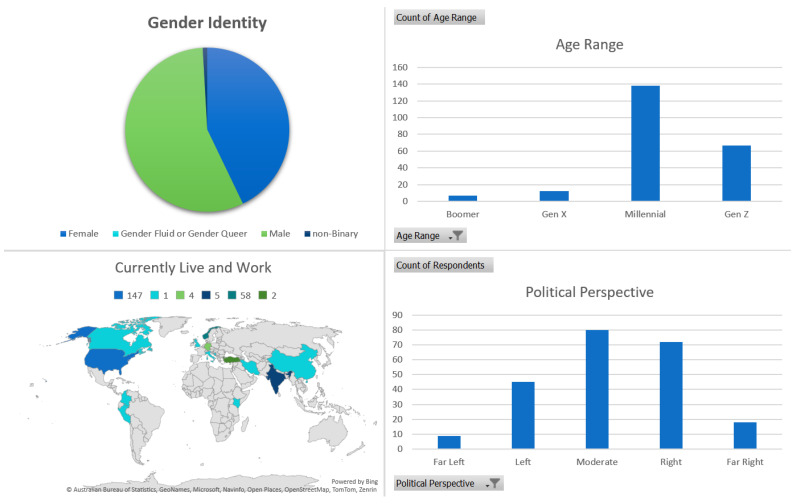
Clockwise from top left: Gender identity of respondents, age range by generational cohort of respondents, the broad political alignment as self-identified by respondents, and the countries identified as where respondents live and work at the time of the survey.

A total of 224 responses were collected in this study. Figure 3 shows the demographic composition of respondents. In this survey, 126 respondents identified as men, 96 as women, and 2 as non-binary. Millennials (138) and Gen Z (67) far outnumbered Boomer (7) and Gen X (12) respondents. Respondents work or live in 11 countries, with 142 respondents coming from the United States, 61 from Norway, 5 each from Germany and India, 3 from Canada, 2 from Türkiye, and 1 respondent each from Australia, Bosnia and Herzegovina, Colombia, Nepal, and Peru. Political perspectives were varied from far left to far right.

### 4.1. What Are the Common Themes That Emerge for Software Engineers About Mental Health?

We specifically targeted software developers who are working in or interested in pursuing a career in digital justice. Respondents were introduced to the concept of digital justice by the textual description: “Does your work relate in some way to ethics, privacy, access to information, use of technology, or some other aspect of marginalized people using technology?” Of our respondents, only 8.9% indicated no desire to work in digital justice or with beneficiaries who use tech for digital justice issues. Of our respondents, 22.8% currently work in or will pursue a career in digital justice. On a three-point scale, the average score for interest in working in digital justice was 1.74.

On the Mental Health Literacy scale, 12.9% of respondents score low, 67.9% scored average, and 19.2% score high. These scores were calculated in alignment with relationship to the standard deviation of responses. The Warwick–Edinburgh calculation for setting high scores and low scores at 15% was employed.

**Figure 4:**
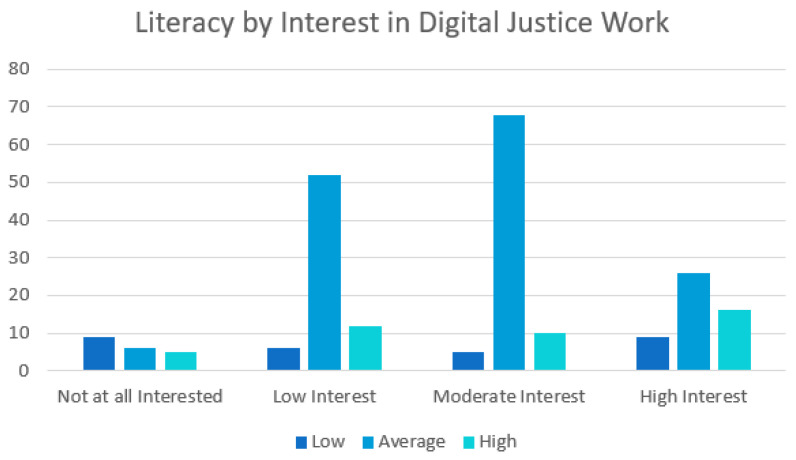
The total number of respondents based on their interest in digital justice work. This is represented by their mental health literacy scores (low, average, and high).

In examining the connections between mental health literacy and digital justice, the results showed the *p*-value to be extremely significant at 0.037 with a 95% confidence interval. Table 1 shows the group, mean, standard deviation, and SEM. Standard error of difference is 0.072. Figure 4 shows the total number of respondents and their interest in digital justice work by mental health literacy score. Thus, an interest in pursuing digital justice work may increase as mental health literacy scores increase.

In total, 673 self-care activities were identified by the survey respondents. Of those, 226 unique self-care activities were identified for an average of three self-care activities per respondent. The top 10 most identified self-care activities can be seen in Table 2.

Table 2 shows the Top 10 self-care activities identified by participants. These activities can be performed either alone or with others.

**Figure 5:**
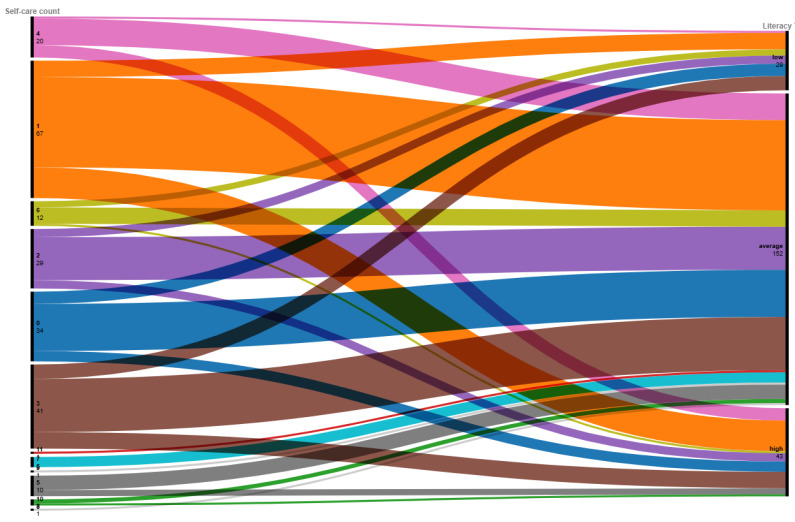
A correlation alluvial plot of the identified number of self-care activities and mental health literacy score. Mentioning more self-care activities does not necessarily equate to higher mental health literacy.

Respondents identified between 0 and 11 activities that they engage in to prioritize their self-care, and the average per respondent is 2.5 activities. Of these listed activities, identifying two activities was correlated with having a high mental health literacy or being able to recognize the connections between mental health symptoms and conditions, treatments, identifying resources, and understanding the scope of normal emotions. However, identifying more activities, particularly nine or more activities, correlated with an average mental health literacy score. This is seen in Figure 5.

**Figure 6:**
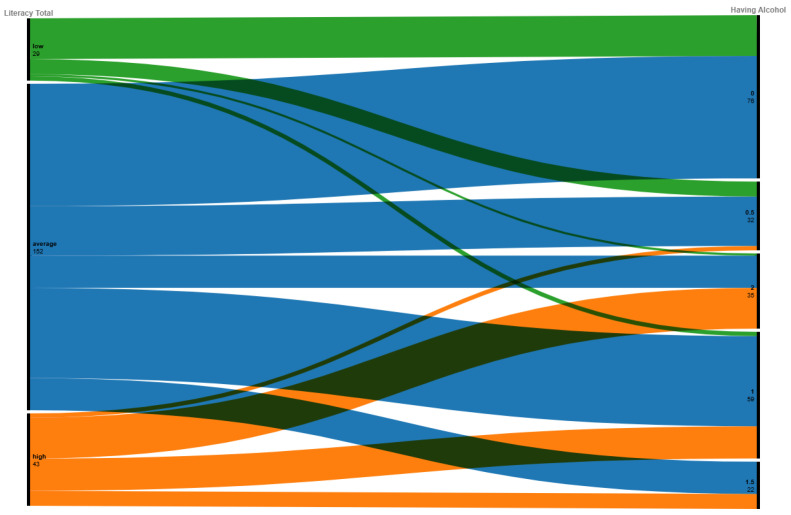
A correlation alluvial plot which shows alcohol identified as a coping strategy for anxiety and depression by mental health literacy score. There is no indication that a higher mental health literacy score indicates knowledge about the use of alcohol.

The data confirmed that having a higher mental health literacy score is correlated to understanding anger, depression, and anxiety as normal human emotions. However, high mental health literacy is not correlated with understanding the use of alcohol consumption. Figure 6 shows that over 90% of respondents with high mental health literacy identified that drinking alcohol to relax is an acceptable treatment for both anxiety and depression. This requires further investigation.

**Figure 7:**
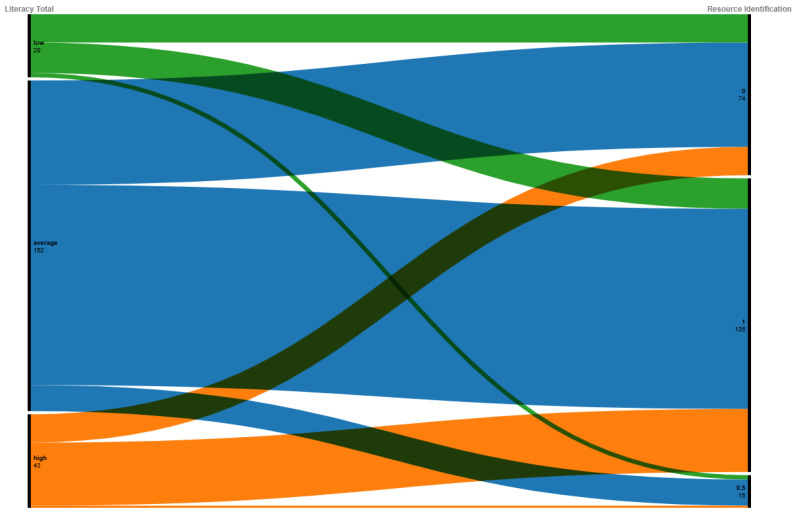
A correlation alluvial plot depicting the connection between mental health literacy score and the ability to identify a resource to support mental health in one’s current area.

In addition, having a high mental health literacy score does not correlate with the ability to find resources in one’s area. Approximately one-third of respondents with high mental health literacy could not identify nor provide a link to a mental health resource in their area, as shown in Figure 7.

### 4.2. To What Extent Are the States of Mental Health in Alignment Between Students and Practitioners in Software Engineering?

First, we present the overall correlations among all the variables computed from the survey. Table 3 presents the correlations. Among the variables that we are focusing on in this contribution, we observe that depression and anxiety have a high positive correlation between them (r(203) = 0.85, *p* < 0.0001).

The dependence analysis for the relation between gender and age range was calculated. A chi-square test of independence showed that the two factors are significantly related (χ^2^ = 5.5, *p* < 0.05). However, none of the residuals are significant (Table 4), and therefore we can consider age and gender distributions in our data independent of each other.

**Figure 8:**
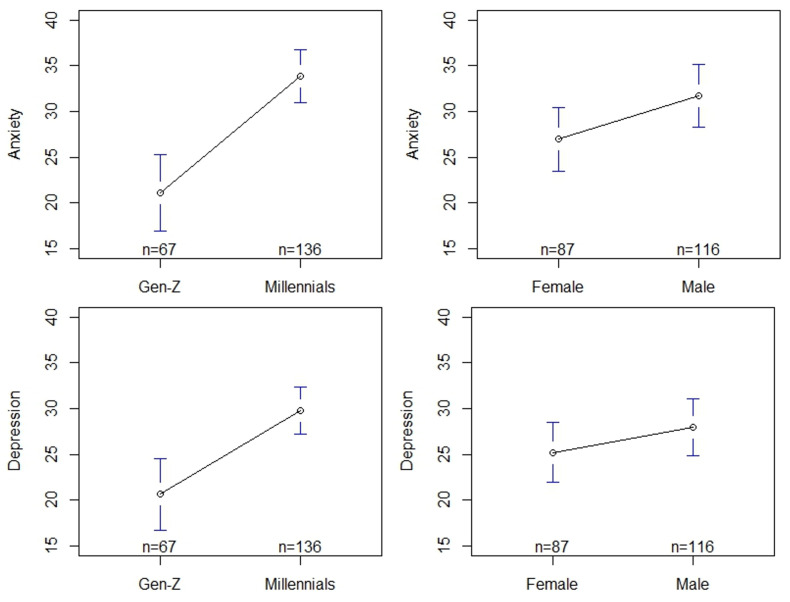
One-way ANOVA mean plots for anxiety and depression for age (**left**) and gender (**right**). The blue bars for Gen-Z, Millennials, Female, and Male in all four images indicate 95% confidence intervals.

Depression and anxiety were compared across genders and age groups (Figure 8). Overall, two separate one-way ANOVA tests, without assuming equal variances among the two groups, showed that there is no difference between males and females concerning anxiety (F[1,196.02] = 3.62, *p* > 0.05) and depression (F[1,192.54] = 1.46, *p* > 0.05). However, we observe differences in anxiety and depression between the two age groups. Two separate one-way ANOVA tests, without assuming equal variances among the two groups, showed that Millennials have higher levels of both anxiety (F[1,130.28] = 25.14, *p* < 0.0001) and depression (F[1,126.72] = 14.89, *p* < 0.0005) than Gen-Z.

### 4.3. What Is the State of the Gender Gap in Anxiety and Depression in Software Engineers Today?

**Figure 9:**
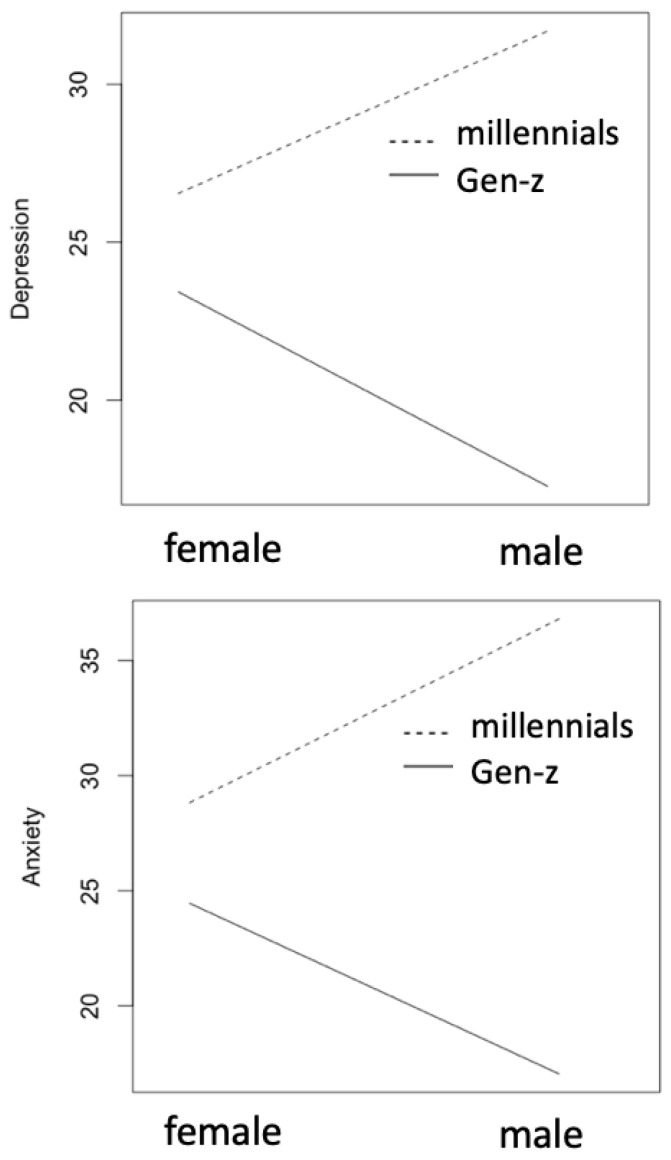
Interaction effect plots for anxiety and depression across the gender and age groups indicate that male Millennials experience higher rates of anxiety and depression that female Millennials.

Finally, we present the interaction effect analysis from comparing depression and anxiety across combined gender and age groups (Figure 9). We observe that male Millennials have higher anxiety than female Millennials whereas, male Gen-Z have lower anxiety than female Gen-Z (F[1,199] = 9.36, *p* < 0.01). We also observe that male Millennials have higher depression than female Millennials, whereas Gen-Z men have lower depression than Gen-Z women (F[1,199] = 5.82, *p* < 0.05).

## 5. Discussion

To address the first research question, we examined the themes around mental health that emerged in the survey. The data show that the number of self-care activities one performs or can recall is not necessarily indicative of mental health literacy. Of the respondents, 3.125% indicated that they do not perform anything intentionally for self-care. While two respondents indicated they no longer have time to perform intentional self-care activities, they were able to recall activities they used to perform to foster resilience.

To address the second research question, we examined the scores of validated instruments used to assess different factors of mental health. Those factors are literacy, self-efficacy, well-being, the affective state of happiness, and symptomatic evaluations of anxiety and depression. The Mental Health Literacy score is a composite score based on a survey designed to identify emotions, recognize symptoms and treatments, and engage with mental health resources. While it may seem obvious that one’s ability to recognize anger and anxiety is connected to their own mental health literacy, this may not be the case. The ability to recognize anxiety and anger as natural human emotions is not necessarily indicative of one’s mental health literacy. The same is true for recognizing symptoms of depression. However, there is some correlation between people with low mental health literacy that cannot identify anxiety but can identify anger as a natural emotion. This is worth further exploration. We have also identified some interesting correlations in the results of the instruments.

Imposter syndrome is generally associated with self-doubt and undervaluing “intellect, skills, or accomplishments among high-achieving individuals”. This includes a lack of internalizing praise or successes and is often accompanied by anxiety, depression, burnout, perfectionism, and a fear of being found out a fraud. Our results indicated a positive correlation between imposter syndrome and anxiety, as well as imposter syndrome and depression. In addition, results for the happiness index and imposter syndrome, happiness index and anxiety, and the happiness index and depression indicate the luck dimension of imposter syndrome. This includes undervaluing successes and accomplishments. Instead of being something worked for and earned, these are instead attributed to being in the right place at the right time, good timing, or being lucky [36].

To examine the third research question relating to the Gender Gap, we examined age and gender in relation to anxiety and depression. In our findings, Gen Z has a higher positive correlation between imposter syndrome and anxiety than Millennials. Gen Z also exhibits a higher positive correlation between imposter syndrome and depression than Millennials. Gen Z also has lower positive correlation between happiness and depression than Millennials. Finally, Gen Z exhibits a higher negative correlation between literacy and anxiety than Millennials.

Our findings differ from those found in previous research. In previous studies, a Gender Gap, where women experience more symptoms than men, was present in both anxiety and depression, and this Gender Gap increased with age. In this study, overall, gender in isolation has no relationship with anxiety or depression. Overall, Gen Z has lower anxiety and depression than Millennials. However, when we combine age and gender, we see two interactions. First, Gen Z women have higher anxiety and depression than Gen Z men, but female Millennials have lower anxiety and depression than male Millennials. While these results reflect these differences in our population, it should be considered that Generation Z will not show the same results when they reach the age of Generation Y.

These results indicate that looking at gender across a wide range of population in terms of their age will not give us enough information about their anxiety and depression levels, but if we combine the gender with a broad sense of the age range, from a generational context, then we gain a deeper insight into experiences and expressions of anxiety and depression. Male Millennials have significantly higher anxiety and depression than Gen Z men; however, this is not true for female Millennials and their Gen Z counterparts. Their anxiety and depression are not statistically significant across the two age ranges. Finally, the Gender Gap is increasing for both anxiety and depression, and it is getting higher for men. This is not evidenced in the literature about the Gender Gap, but it is worth further exploration.

### 5.1. Limitations

This study has limitations. First, because this is a correlational study, generalizability is low. Also, including two populations usually kept separate and distinct, namely practitioners and students, also limits generalizablility. Moreover, Gen Z may know more about mental health and self-care than Millennials based on the prevalence of information about these in social media channels. Even though the population for this study is software engineers in the software engineering pipeline, these considerations should be taken under consideration when evaluating these results.

### 5.2. Future Studies

The connections between self-care and mental health will be further explored using other demographic data. We gathered the political perspectives, country of work, and country of residence, all of which may yield other interesting results about the current state of mental health in software engineers important to advancing responsible SE. We also gathered information about respondent roles in the software engineering pipeline, mental health support services in the workplace, and general awareness of mental health and self-care. All of this information will be used to design interventions for software engineers to address issues related to well-being and resilience.

Two people, as noted in the demographic breakdown of respondents, did identify as non-binary. Targeting non-binary emerging adults along the software engineering pipeline is a planned direction. In order to not commit the erasure of non-binary people in the future, they need to be a targeted population of study.

Replicating the study using professional networks is recommended. This can serve as a comparative analysis. Such professional organizations and venues include ACM SIGSOFT, IEEE Software Community, and LinkedIn. This better distribution of professionals will enhance results along that part of the pipeline. This will also help validate our findings about the differences in the Gender Gap in depression and the need for men to be a focus group for mental health awareness, discussion, and support services.

### 5.3. Threats to Validity

The threats to validity include the choice of statistical analyses and the absence of correction for *p*-values. While some may question the enmeshment of the sample, we show that the population is justified based on our examination from an age perspective. To address these threats to validity, the population was split into student and non-student, and analyses were calculated in order to account for possible confounding variables. Confidence intervals and required sample size for generalizability were calculated.

### 5.4. Replication Package

See the Replication Package, which is available in the Open Science Framework at https://doi.org/10.17605/OSF.IO/J5W8M, accessed on 6 February 2024.

## 6. Conclusions

This study investigated the well-being of software engineers in three ways. First, we examined connections between self-care activities and mental health literacy. Additionally, we employed validated assessment tools to scrutinize the current mental health status among individuals engaged in software development, seeking to identify emotional states and indicators of mental health issues. A specific inquiry was dedicated to scrutinizing the Gender Gap concerning anxiety and depression prevalent among software developers today.

Our findings underscore the need for further exploration of the link between mental health literacy and well-being. Moreover, we emphasize the importance of ongoing support not only for women in the workplace but also for men who might be experiencing symptoms of anxiety and depression yet not openly discussing these issues. We also acknowledge that trans and non-binary folks need additional support and recognition for their mental health, well-being, and work in designing the built environment. Here is what those in the software engineering pipeline should do to address our findings:

- Create awareness of self-care activities;
- Explore new techniques for safely exploring self-care together;
- Make help visible;
- Reduce stigmatization for marginalized groups and mental health in general;
- Encourage males to talk about their mental health.

Our study highlights the intrinsic connection between our well-being, resilience, and self-perception, particularly within the field of software development, where individuals also serve as advocates for social justice at various stages of the software development pipeline. Because mental health literacy highly correlates with one’s desire to work in digital justice, educators should prepare these students for compound burnout. Remembering that high mental health literacy does not mean awareness of mental health issues will allow universities and workplaces to create experiences and safe spaces for everyone contributing to the built environment to talk about anxiety, depression, and burnout.

## Figures and Tables

**Table 1:** Descriptive statistics and *t*-test results depicting the connection between an interest in working in digital justice and mental healthy literacy score as mean, standard deviation (SD), standard error of the mean (SEM), and number of responses (N).

*T*-Test Results for Mental Health Literacy and an Interest in Working in Digital Justice		
**Group**	**Digital Justice Work**	**Mental Health Literacy**
Mean	0.71	1.06
SD	0.46	0.56
SEM	0.03	0.04
N	224	224
*p*-value	<0.0001	Extremely Significant

**Table 2:** A depiction of the top ten self-care activities identified by respondents and their corresponding total mentions.

Top 10 Self-Care Activities	
**What Do You Do for Self-Care?**	**Total**
Reading	56
Exercising and working out (gym, endurance, strength, lift weights)	48
Playing games	26
Eating well	23
Dancing	20
Chatting with friends	19
Yoga	19
Hiking	18
Sleeping well	17
Therapy	12
Walking	12

**Table 3:** Overall correlations among the different variables from the survey. WB = well-being; LL = literacy level; IS = imposter syndrome; SE = self-efficacy; HP = happiness index; AX = anxiety; DP = depression; NS = not significant correlations. All the correlations presented are significant with *p* < 0.05.

Dependence Analysis of Mental Health Assessments						
	**LL**	**IS**	**SE**	**HP**	**AX**	**DP**
**WB**	NS	NS	0.76	0.59	NS	NS
**LL**	-	−0.38	NS	−0.22	−0.48	−0.42
**IS**	-	-	NS	0.35	0.63	0.59
**SE**	-	-	-	0.74	0.20	NS
**HP**	-	-	-	-	0.56	0.45
**AX**	-	-	-	-	-	0.85

**Table 4:** Cross-tabulation of gender and age groups. The numbers in parentheses are the chi-square residuals. The values greater than 1.96 and smaller than −1.96 are considered significant.

Chi-Square Results for Gender and Age by Generational Cohort		
	**Millennials**	**Gen-z**
**Female**	50 (−1.08)	37 (1.54)
**Male**	86 (0.93)	30 (−1.33)

## Data Availability

See the Replication Package, which is available in the Open Science Framework at https://doi.org/10.17605/OSF.IO/J5W8M, accessed on 6 February 2024.
